# Pericytes’ Circadian Clock Affects Endothelial Cells’ Synchronization and Angiogenesis in a 3D Tissue Engineered Scaffold

**DOI:** 10.3389/fphar.2022.867070

**Published:** 2022-03-21

**Authors:** Valeria Mastrullo, Daan R. van der Veen, Priyanka Gupta, Rolando S. Matos, Jonathan D. Johnston, John H. McVey, Paolo Madeddu, Eirini G. Velliou, Paola Campagnolo

**Affiliations:** ^1^ Cardiovascular Section, Department of Biochemical Sciences, University of Surrey, Guildford, United Kingdom; ^2^ Chronobiology Section, Department of Biochemical Sciences, University of Surrey, Guildford, United Kingdom; ^3^ Bioprocess and Biochemical Engineering Group (BioProChem), Department of Chemical and Process Engineering, University of Surrey, Guildford, United Kingdom; ^4^ Experimental Cardiovascular Medicine, University of Bristol, Bristol Heart Institute, Bristol Royal Infirmary, Bristol, United Kingdom; ^5^ Centre for 3D Models of Health and Disease, Department of Targeted Intervention, Division of Surgery and Interventional Science, University College London (UCL), London, United Kingdom

**Keywords:** circadian, angiogenesis, vasculature, pericytes, tissue engineering and regenerative medicine

## Abstract

Angiogenesis, the formation of new capillaries from existing ones, is a fundamental process in regenerative medicine and tissue engineering. While it is known to be affected by circadian rhythms *in vivo*, its peripheral regulation within the vasculature and the role it performs in regulating the interplay between vascular cells have not yet been investigated. Peripheral clocks within the vasculature have been described in the endothelium and in smooth muscle cells. However, to date, scarce evidence has been presented regarding pericytes, a perivascular cell population deeply involved in the regulation of angiogenesis and vessel maturation, as well as endothelial function and homeostasis. More crucially, pericytes are also a promising source of cells for cell therapy and tissue engineering. Here, we established that human primary pericytes express key circadian genes and proteins in a rhythmic fashion upon synchronization. Conversely, we did not detect the same patterns in cultured endothelial cells. In line with these results, pericytes’ viability was disproportionately affected by circadian cycle disruption, as compared to endothelial cells. Interestingly, endothelial cells’ rhythm could be induced following exposure to synchronized pericytes in a contact co-culture. We propose that this mechanism could be linked to the altered release/uptake pattern of lactate, a known mediator of cell-cell interaction which was specifically altered in pericytes by the knockout of the key circadian regulator *Bmal1*. In an angiogenesis assay, the maturation of vessel-like structures was affected only when both endothelial cells and pericytes did not express *Bmal1*, indicating a compensation system. In a 3D tissue engineering scaffold, a synchronized clock supported a more structured organization of cells around the scaffold pores, and a maturation of vascular structures. Our results demonstrate that pericytes play a critical role in regulating the circadian rhythms in endothelial cells, and that silencing this system disproportionately affects their pro-angiogenic function. Particularly, in the context of tissue engineering and regenerative medicine, considering the effect of circadian rhythms may be critical for the development of mature vascular structures and to obtain the maximal reparative effect.

## 1 Introduction

Circadian rhythms are essential regulators of the physiology of many biological processes (Lowrey and Takahashi, 2004). Every organ and possibly every nucleated cell of the body possess an endogenous molecular clock machinery, that in a healthy individual is synchronized by the main light-entrained pacemaker in the hypothalamus, or non-photic timing cues, such as feeding (Buhr and Takahashi, 2013). The molecular clock relies on a negative feedback loop mechanism, where Aryl Hydrocarbon Receptor Nuclear Translocator Like (ARNTL or BMAL1) and Clock Circadian Regulator (CLOCK) heterodimer functionality is inhibited by Period (PER1 - 3) and Cryptochrome (CRY 1,2) multimeric complexes (Lowrey and Takahashi, 2004; Buhr and Takahashi, 2013). A second feedback loop involves Nuclear Receptor Subfamily 1 Group D Member 1 (NR1D1 or REV-ERBα) and Retinoic Acid Receptor-Related Orphan Receptor (ROR) elements that represses and enhances *Bmal1* transcription, respectively (Buhr and Takahashi, 2013). Importantly, *Bmal1* is the only non-redundant gene in the core circadian clock, making it a key element in the manipulation of molecular clocks *in vitro* (van der Horst et al., 1999; Bae et al., 2001; Haque et al., 2019).

In the vasculature, the existence of a clock has been studied in zebrafish (Jensen and Cao, 2013) and mice (Anea et al., 2009; Bhatwadekar et al., 2017), where its functionality has been correlated to a healthy angiogenesis (Jensen et al., 2014). Angiogenesis, the formation of new capillaries from existing ones, is a multifactorial process that requires synchrony between endothelial cells and pericytes to develop new functional blood vessels (Mastrullo et al., 2020). In both animals and humans, the effect of the disruption of circadian rhythms on the endothelial function has been established, affecting for instance vascular tone, blood pressure and heart rate (Panza et al., 1991; Viswambharan et al., 2007; Bhatwadekar et al., 2017; Crnko et al., 2018; Rodrigo and Herbert, 2018; Han et al., 2021); however, the molecular mechanism underlining the drivers of the vasculature clock has not yet been defined. Within the cardiovascular system, molecular clocks have been described in endothelial cells (Takeda et al., 2007; Westgate et al., 2008), and smooth muscle cells (Nonaka et al., 2001), but no study has specifically investigated its presence in pericytes. Pericytes exist within the microvasculature of all organs and tissues in the human body. They possess pro-angiogenic potential and form a resident mesenchymal progenitor niche within the tissues (Yamazaki and Mukouyama, 2018). While the existence of a clock in other non-vascular mesenchymal populations has been identified (Huang et al., 2009), the information on the pericytes’ own is scarce.

A contact-mediated and paracrine cross-communication between endothelial cells and pericytes favors the correct development of new blood vessels, as well as the maintenance of the existing ones (Gerhardt and Betsholtz, 2003; Armulik et al., 2005; Chatterjee and Naik, 2012). Many factors have been described to mediate this cross-communication, including growth factors (VEGF, PDGF, Ang-1/-2), micro-vesicles and adhesion molecules (Carmeliet and Jain, 2011). However, the role of circadian rhythms in the endothelial/pericyte crosstalk and the capacity of pericytes to influence endothelial cells’ circadian clock are largely unknown.

Angiogenesis does not occur frequently in adult organisms and is mainly limited to neoplastic growth and post-injury repair. In regenerative medicine, however, the induction of efficient and mature vascularization is crucial to drive tissue regeneration (Mastrullo et al., 2020). Human primary pericytes have been successfully used as a source of reparative cells for cell therapy of ischemic tissues (Campagnolo et al., 2010; Katare et al., 2011). Similarly, vascularization of tissue engineered construct remains an unsolved challenge to deliver large size synthetic solutions for organ and tissue replacement (Mastrullo et al., 2020). In these contexts, a further understanding of the mechanisms underlining the angiogenic cell-to-cell communication is necessary to advance in the field and further investigation is warranted to determine the role of circadian genes in angiogenesis (Mastrullo et al., 2020).

In this paper, we describe the existence of a molecular circadian clock in human primary pericytes, and the negative implication of the disruption of the molecular circadian clock on their pro-angiogenic crosstalk with endothelial cells in 3D vascularization. Furthermore, we describe a regulatory role for pericytes in synchronizing the endothelial’s clock and a potential contribution of lactate in this process. This work suggests that the circadian clock should be considered in the context of regenerative medicine and tissue engineering, and that the synchronization of endothelial function may in part depend on local signals from perivascular cells.

## 2 Materials and Methods

### 2.1 Cell Culture

Human umbilical vein endothelial cells (HUVEC) were purchased from Promocell, grown, and expanded in endothelial growth media-2 (EGM-2, Promocell, Germany). Primary human saphenous vein pericytes (SVP) were kindly donated from Prof Madeddu (University of Bristol) and cultured in EGM-2. SVP were grown and expanded in flasks or 35 mm dishes pre-coated with 0.1% fibronectin (Merck, United States) and 0.05% gelatin (Merck, United States) solution. Cells were maintained at 37°C with 5% CO_2_, passaged at 80% confluency and used up to passage 8 for experiments.

### 2.2 RNA Isolation, cDNA Synthesis and RT-qPCR

For gene expression experiments, cells were grown overnight in 6-well plates until confluency (90–100%) was reached. Confluent plates were used for all experiments to exclude the cell cycle as a potential cause of any patterns in gene expression that might be observed over the time course. Confluent monolayers were exposed to growth media supplemented with 50% FBS for 2 h, at 37°C with 5% CO_2_, to synchronize the circadian clock between cells. The start of the 2 h serum shock was considered time ZT0 (Zeitgeber time 0), after which total RNA was collected every 4 h for 36 h (N = 3, n = 9). The results from the first 8 h were excluded from the analysis to avoid the immediate effect of the treatment with high serum concentration on the cells. RNA was isolated using Quick-RNA Miniprep (Zymo Research, United States). The cell lysis buffer was added directly to the dish at the designated time point and RNA was extracted following the manufacturer’s protocol. All samples were quantified using NanoDrop to determine RNA concentration and purity. Single-strand cDNA was synthesized using QuantiTect Reverse Transcription Kit (Qiagen, United Kingdom). Real-time quantitative PCR was conducted using the PowerUp SYBR Green Master Mix (Applied Biosystems, United States). Samples were analyzed on QuantStudio7 Flex Real-Time PCR System (Applied Biosystems, United States). The 2^−ΔΔCt^ method was used to calculate the relative fold gene expression for each sample. Briefly, the triplicate Ct values of the target genes and the β-actin (housekeeping gene) were averaged, the first Δ was calculated by subtracting the average Ct value of the target gene to the average Ct value of β-actin; the second Δ was calculated by subtracting the ΔCt of samples collected at time points to the ΔCt of the ZT0 sample. Finally, the fold gene expression was calculated by doing the 2 to the power of negative ∆∆Ct.

All primers listed in Table 1 were designed using human sequences on the Primer Design tool (NCBI-NIH) and tested for the correct amplicon length in the SVP cells by PCR using GoTaq Green Mastermix (Promega, United Kingdom).

**TABLE 1:** List of primers used for mRNA expression of clock genes in human cells.

Gene	GenBank	Forward primer (5′-3′)	Reverse primer (5′-3′)
*Bmal1*	NM_001030272	TGG​ATG​AAG​ACA​ACG​AAC​CA	TAG​CTG​TTG​CCC​TCT​GGT​CT
*Clock*	NM_001267843	GCAGCAGCAGCAGCAGAG	CAG​CAG​AGA​GAA​TGA​GTT​GAG​TTG
*Per2*	NM_022817	GAC​ATG​AGA​CCA​ACG​AAA​ACT​GC	AGG​CTA​AAG​GTA​TCT​GGA​CTC​TG
*Rev-erbα*	NM_021724	TGC​TGC​AGG​GTG​CTT​CGG​AT	TAG​GTG​ATG​ACG​CCA​CCT​GTG​T
*β-Actin*	NM_001101.4	AGA​GCT​ACG​AGC​TGC​CTG​AC	AGC​ACT​GTG​TTG​GCG​TAC​AG

### 2.3 Quantitative Immunofluorescence

For protein quantification, cells were grown on 13 mm diameter glass coverslips. Confluent cells were exposed to 50% FBS growth media for 2 h, at 37°C with 5% CO_2_, to synchronize their circadian clock. This was considered time ZT0, after which cells were collected every 4 h and immediately fixed in 4% paraformaldehyde (PFA, ThermoFisher Scientific, United States) for 20 min at room temperature. Immunofluorescence staining was performed using PBS with 4% FBS as blocking agent and antibodies anti-ARNTL produced in rabbit (1:200, Merck, United States) or anti-NR1D1 produced in rabbit (1:200 μg/μl, Merck, United States), followed by AlexaFluor goat anti-rabbit 488 (1:200, ThermoFisher Scientific, United States) secondary antibodies. Phalloidin-iFluor 594 Reagent (Abcam,United Kingdom) was used to stain the actin filaments and DAPI (4′,6-diamidino-2-phenylindole, 1:1,000, Sigma) was used for nuclear staining. The stained slides were imaged using the 20x objective on the Nikon Eclipse. Images were processed using the NIS Elements software (Nikon) and analyzed using the software ImageJ. For each image, a two-step method was developed. First, individual nuclei were determined based on a user-defined threshold. These nuclear signals were converted in ROI (region of interest) sets containing all nuclei and Integrated Density (IntDen) of FITC signal was quantified within individual nuclei. For each time point, a technical duplicate was performed for each of the three biological replicates (*N* = 3, *n* = 6). Data were plotted, and a cosinor curve fitting was performed using the software GraphPad Prism v.8.0.

### 2.4 Lentivirus Production and Viral Transduction

Promoter sequences of three clock genes (*Bmal1*, *Per2* and *Rev-erbα*; Table 2) were identified from human genome database and purified by PCR from the DNA of a human donor. Plasmids were cloned using the Gateway Cloning technology (ThermoFisher, United States) and using the final plasmid backbone pLNT-GW-JDG, kindly donated by the Prof Waddington’s group, to produce bioluminescent reporters (Buckley et al., 2015). For the lentivirus production, HEK-293FT producer cells (Gibco, United States) were transfected with 3 plasmids: plasmid of interest (carrying *Bmal1*, *Per2* or *Rev-erbα* promoter), pMD2. G VSVg env (Addgene, United States) and pCMVR8,74 packaging (Addgene, United States). Lentiviral particles were collected from the cell culture supernatants, concentrated by ultracentrifugation, and immediately stored at −80°C. Lentiviral particles titration was performed using p24 ELISA kit (Takara Bio, United States). SVP and HUVEC were transduced with 10 multiplicity of infection (MOI) and 5 MOI of lentiviral vectors, respectively. Fresh media was added after 24 h to replace the lentivirus-containing growth media.

**TABLE 2:** List of primers used for the purification of human promoters from human DNA.

Gene	Forward primer (5′-3′)	Reverse primer (5′-3′)
*Bmal1*	ACC​CAG​AGA​AGA​GGG​ACA​TC	CTC​CGT​CCC​TGA​CCT​ACT​TT
*Per2*	TGA​GGG​CGT​AGT​GAA​TGG​AAG	TGT​CAC​CGC​AGT​TCA​AAC​GA
*Rev-erbα*	ATC​TAC​ATG​TTC​CCC​TCT​GAG​TAG​T	TAT​TTC​ACT​CTG​CCA​ATC​TCA​GCC

### 2.5 Bioluminescence

For bioluminescence recordings, cells that were transduced with the bioluminescence reporters were grown overnight into 35 mm dishes until confluency (90–100%) was reached. Confluent cells were exposed to 50% FBS growth media for 2 h, at 37°C with 5% CO_2_, to synchronize the circadian clock. Cells were washed twice with Dulbecco’s phosphate buffered saline (D-PBS) and the recording media was added to the dish. The recording media contained phenol-red free EGM-2 (Promocell, Germany) with 0.1 mM endotoxin-free Luciferin (Promega,United Kingdom) and 20 mM HEPES (pH 7.4). Culture dishes were sealed with high-vacuum grease and 40 mm glass coverslips (VWR, United Kingdom). Bioluminescence signals were recorded in the LumiCycle device (Actimetrics, United States) and baseline subtraction on running average measurements was performed by using the Lumicycle Analyzer software. A damped sin wave (non-linear regression) with a 24 h wavelength was superimposed on bioluminescence reports, using GraphPad Prism 8.0.

#### 2.5.1 Contact and Non-contact Co-cultures

For contact co-cultures, 6 × 10^4^ SVP were grown overnight into 35 mm dishes and synchronized with a single pulse of 100 nM dexamethasone (Merck, United States) for 20 min. The start of the dexamethasone treatment was considered time ZT0. *Bmal1*:Luc HUVEC were detached, counted, and 2 × 10^5^ cells were seeded on top of synchronized SVP for bioluminescence recordings (*N* = 3, *n* = 11).

For non-contact co-cultures, 4 × 10^5^ *Bmal1*:Luc HUVEC were grown overnight on 6-well inserts with 0.4 μm PET-membrane (Merck, United States). The following day, the inserts were gently placed in 35 mm dishes containing confluent SVP, synchronized as previously described (*N* = 3, *n* = 6).

### 2.6 shRNA BMAL1 Knockdown

Lentiviral particles containing shRNA for BMAL1 knockdown were purchased from Merck (TRCN0000331078, Sequence: CCG​GGC​AAC​AGC​TAT​AGT​ATC​AAA​GCT​CGA​GCT​TTG​A-TAC​TAT​AGC​TGT​TGC​TTT​TTG). MISSION^®^ pLKO.1-puro Non-Mammalian shRNA Control Plasmid DNA was purchased from Merck (SHC002) and used to produce lentiviral particles from HEK-293FT producer cells, as described above. HUVEC and SVP were transduced with 10 MOI of lentivirus and stably transduced clones were selected using 4 μg/ml Puromycin dihydrochloride (Merck, United States). Viability/apoptosis were measured with the ApoTox Glo assay (Promega, United Kingdom), following manufacturer’s protocol. Briefly, 2 × 10^4^ cells were plated in a black clear-bottom 96well plate and viability/apoptosis was assessed 24, 48, 72 and 96 h after plating (for each time point *N* = 1; *n* = 4).

### 2.7 Lactate Release and Glucose Intake Measurements

1.5 × 10^4^ HUVEC and 0.5 × 10^4^ shNEG/BMAL1 SVP were cultured separately or co-cultured in a ratio 3:1 in wells of a 96-well plate and allowed to attach overnight. The following day, co-cultures were synchronized with a single pulse of 100 nM dexamethasone, as previously described, and an aliquot of 5 µl of culture supernatant was collected every 4 h for downstream analysis. Lactate release and glucose intake were measured using Lactate-Glo (Promega, United Kingdom) and Glucose-Glo (Promega, United Kingdom) assays respectively, following manufacturer’s protocols. Supernatants were diluted 1:50 for lactate and 1:200 for glucose analysis.

### 2.8 Matrigel Assay

For the Matrigel assay shNEG/shBMAL1 HUVEC and shNEG/shBMAL1 SVP cells were detached and counted. For the single culture of shNEG/shBMAL1 HUVEC, a total number of 3 × 10^4^ cells was used, while for the co-cultures, the ratio between HUVEC:SVP was 3:1 to obtain 3 × 10^4^ total number of cells. 30 µl of growth factor-reduced Matrigel (Corning, United States) was dispensed in each well of a 96-well plate and allowed to solidify. Three bright-field images were taken from each well after 8 h. Three biological replicates, and 3 technical replicates were performed for each experiment (*N* = 3, *n* = 9). Image analysis was performed using ImageJ software and applying the macro “Angiogenesis analyzer” (Carpentier et al., 2020) with additional integrated features from OCTAVA toolbox (Untracht et al., 2021).

### 2.9 Polyurethane Scaffold Preparation

Polyurethane (PU) scaffold was fabricated as previously described (Velliou et al., 2015; Totti et al., 2018; Gupta et al., 2019; Gupta et al., 2020) by thermal induced phase separation (TIPS) method. The generated scaffolds are highly porous, with interconnected pores of an average size of 120–140 μm. The scaffolds were cut in blocks of dimensions 2.5 mm × 2.5 mm x 5 mm. The scaffolds were sterilized with 70% ethanol overnight and exposure to dim UV-light for 40 min. Post-sterilization, the scaffolds were surface modified with fibronectin/gelatin via physisorption. Briefly, the scaffolds were immersed in 0.1% fibronectin and 0.05% gelatin solution, centrifuged for 20 min and placed in wells of a 48-well plates on top of a polydimethylsiloxane (PDMS) disks. PDMS is a hydrophobic material chosen for reducing the wash-out effect after cell seeding.

### 2.10 3D Cell Culture and Synchronization

5 × 10^5^ HUVEC, 2.5 × 10^5^ SVP or a total of 5 × 10^5^ cells (HUVEC:SVP, 3:1) were suspended in 30 µl of EGM-2 and seeded on each scaffold. The scaffolds were pinned to the PDMS disks using entomology pins, and fresh media was added in each well of a 48-well plate. Scaffolds were incubated at 37°C with 5% CO_2_. For the synchronization experiments, a single pulse of 100 nM dexamethasone was added to the 48-well plates for 2 h, to allow a proper absorption of the synchronizing agent into the scaffold thickness. The scaffolds were washed with PBS and incubated in normal growth media for 2,7,10 or 14 days (N = 3 per each condition). At each time point, the scaffolds were immersed in 4% PFA for 30 min and then washed 3 times with PBS.

### 2.11 OCT Embedding, Sectioning and Scaffold Immunofluorescence

The scaffolds were embedded in OCT compound (Agar Scientific, United Kingdom), snap-frozen in cold iso-pentane and kept at −80°C. Cryosections were obtained using the cryostat Zeiss Hyrax (20 μm thickness) on SuperFrost adhesion slides (FisherScientific, United States). Immunofluorescence staining was performed using 20% goat serum (Merck, United States) to block non-specific binding sites. Antibodies anti-CD31 produced in mouse (0.01 μg/μl, ThermoFisher, United States) and anti-NG2 produced in rabbit (0.01 μg/μl, EMD Millipore, United Kingdom) followed by AlexaFluor secondary antibodies produced in goat (1:200, ThermoFisher Scientific, United States) were used to stain endothelial cells and pericytes respectively. DAPI was used to counterstain the nuclei.

#### 2.11.1 Scaffold Cell Distribution Analysis

Four sections for each scaffold were imaged using 10x objective on the Nikon Eclipse inverted fluorescence microscope (Nikon) fitted with lasers and filter blocks for the detection of blue fluorescent signal from DAPI (365 nm) to image the nuclei and the brightfield to image the scaffold structure (*N* = 3, *n* = 12). For each image, the edge of the scaffold was identified, and a line was drawn following the perimeter. Using the “Find Maxima” function in ImageJ, the total number of cell nuclei and the corresponding coordinates were measured. Based on the relative distance from the marked boundaries of the scaffold, the relative frequency of nuclei normalized by the total cell count has been obtained. Heat maps were created using GraphPad Prism 8.0, where each colored band represents the median value. To analyze the cell coverage of the scaffold pores, three images from each scaffold were taken with Nikon confocal microscope (*N* = 3, *n* = 9). The number of CD31 positive cells (endothelial cells) and NG2 positive cells (pericytes) in each pore were counted manually. Moreover, for each pore, the perimeter length, and the coverage was calculated with the software ImageJ.

### 2.12 Statistical Analysis

All graphs were plotted using GraphPad Prism 8.0 Software. Values are reported as mean ± SEM. For the analysis of circadian rhythms, Cosinor fit was performed on GraphPad Prism 8.0, using equation Y=(Mesor + Amplitude*cos (((2*pi)*(X-Acrophase))/Period)) where the initial values to be fit were calculated as follows: Mesor = average of all values; Amplitude = variance of all values; Acrophase = Ymax value. The *p* value of the fit was obtained by comparing the non-linear regression fit to a straight line. For all the other statistical analysis, a 2-way ANOVA with Greiner-Greenhouse correction and Sidak multiple comparison test was performed.

## 3 Results

### 3.1 Serum Shock Synchronizes the Molecular Clock in Human Primary Pericytes

We exposed human primary pericytes to a 50% serum concentration for 2 h (serum shock) and measured the expression of circadian molecules using real-time PCR, immunofluorescence, and reporter cell lines.

**FIGURE 1:**
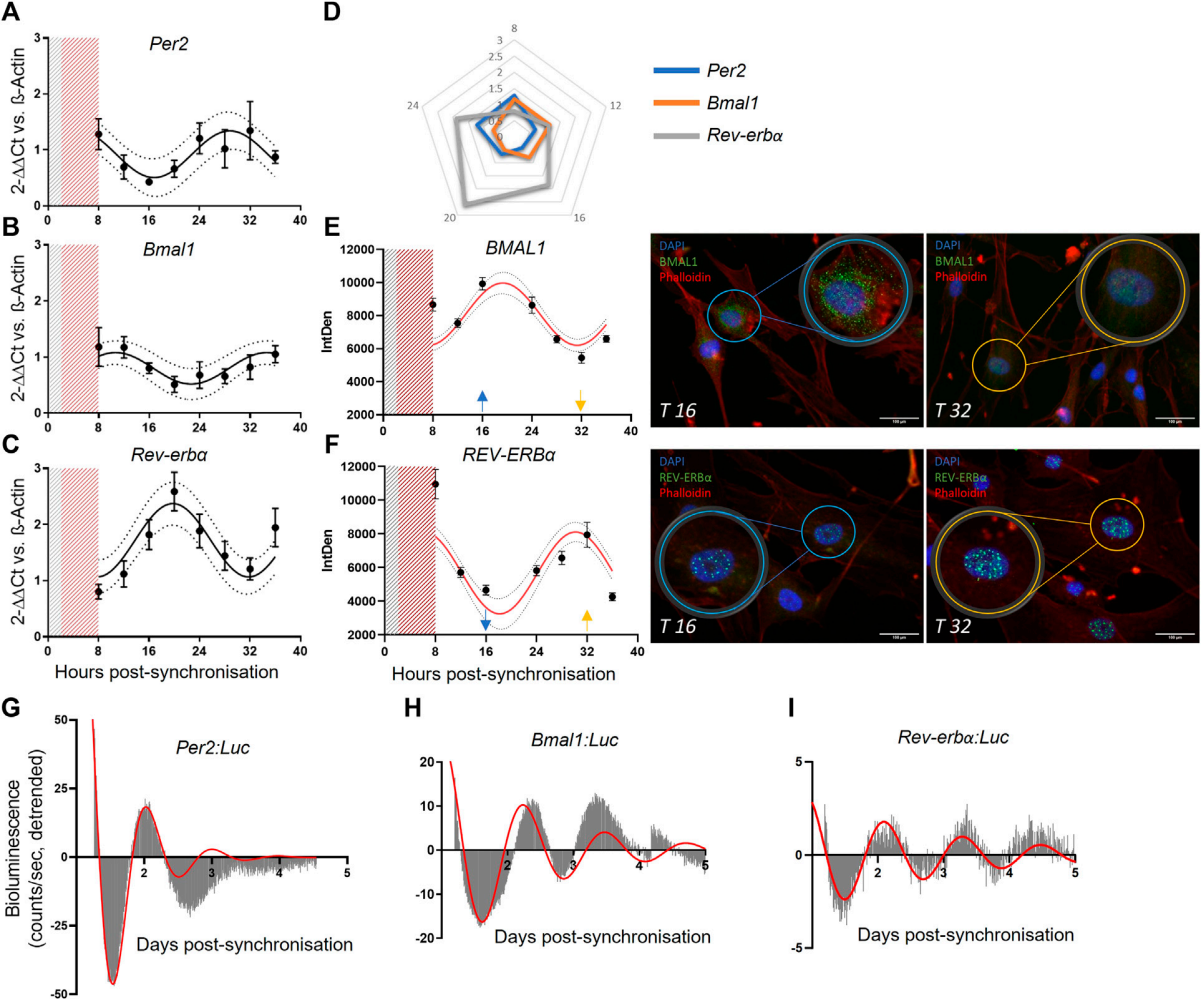
Saphenous vein derived pericytes (SVP) possess an endogenous molecular clock. mRNA expression of Per2 (N = 3, **(A)**), Bmal1 (N = 3, **(B)**) and Rev-erbα (N = 3, **(C)**), in SVP cells. Data is presented as 2^−ΔΔCt^ and normalized on β-actin housekeeping mRNA expression. Error bars represent SEM and 95% CI is shown in the graph. Non-linear regression (Cosinor Curve) is over-imposed and favorably compared against straight line. Radar graph denoting the peak of mRNA expression of each gene over 24 h **(D)**. Immunofluorescence representative images (T16 and T32) and integrated density quantification of nuclear signal intensity of proteins BMAL1 (N = 3, **(E)**) and REV-ERBα (N = 3, **(F)**). Error bars represent SEM and 95% CI are shown in the graph. Non-linear regression (Cosinor Curve, fixed period of 24 h) is over-imposed and favorably compared against straight line (p < 0.05). DAPI blue, Phalloidin red, BMAL11/REV-ERBα green, scale bar: 100 µm. Average detrended oscillatory profile of serum shock synchronized SVP cells transduced with Per2:Luc (N = 2, **(G)**) Bmal1:Luc (N = 2, **(H)**) or Rev-erbα:Luc (N = 2, **(I)**) lentivectors. Red line represents a damped sin wave with a period of 24 h.

Results revealed that the mRNA expression of *Per2*, *Bmal1* and *Rev-erbα* present rhythmicity in human pericytes (Figures 1A–C; N = 3; mean ± SEM and 95% confidence interval shown) peaking approximately at 8, 12 and 18 h after the serum shock, respectively. As expected, *Bmal1* and *Per2* expression patterns were in antiphase, in line with their reciprocal roles in the circadian regulatory feedback loop (Figure 1D). Cosinor fit analysis was performed, and statistical analysis indicated that all the genes analyzed presented a rhythmic profile, with a period close to the expected 24 h (*Per2 p* ≤ 0.005, Period = 24.29 h; *Bmal1 p* ≤ 0.01, Period = 22.95 h; Rev-erbα *p* < 0.0001, Period = 21.82 h).

To confirm that the gene expression results translated into protein expression, we performed immunofluorescence staining at the same time points, and measured the nuclear intensity of each protein. Results showed that BMAL1 and REV-ERBα accumulation in the nuclei was rhythmic after serum shock (Figures 1E,F). Cosinor fit analysis and statistical analysis was performed to identify the rhythmic profile (N = 3; BMAL1 *p* ≤ 0.05; REV-ERBα *p* < 0.0001). The protein quantification data shows a delay of 8 h in relation to the RNA rhythms, in line with the delay expected for protein translation. In addition, we confirmed our findings with bioluminescence assays. We generated lentiviruses carrying the human promoters of *Per2*, *Bmal1,* and *Rev-erbα* upstream the luciferase reporter gene, and transduced primary pericytes. Upon synchronization, the promoter bioluminescence activity was assessed by Lumicycle with a 10 min interval between measurements. Recordings showed that human primary pericytes’ promoter activation of *Per2*, *Bmal1* and *Rev-erbα* was rhythmic up to 4 days of culture (Figures 1G–I; *N* = 2; damped sin wave with fixed 24 h period fitted).

Taken together, these findings indicate that human vascular pericytes display a robust, rhythmic expression of clock genes measured at both gene and protein level, with a synchronicity that is in line with their known functionality.

### 3.2 Endothelial Cells Do Not Display Circadian Rhythmicity *in vitro* but Can Be Induced by Contact Co-culture With Pericytes

Endothelial cells are key players in vascular homeostasis and many *in vivo* studies have identified a rhythmicity in their function (Anea et al., 2009; Wehrens et al., 2012; Shang et al., 2016; Crnko et al., 2018). However, the precise signals driving the synchronization of endothelial cell function are not completely understood, and in particular the role of the closely associated pericytes has not been evaluated.

**FIGURE 2:**
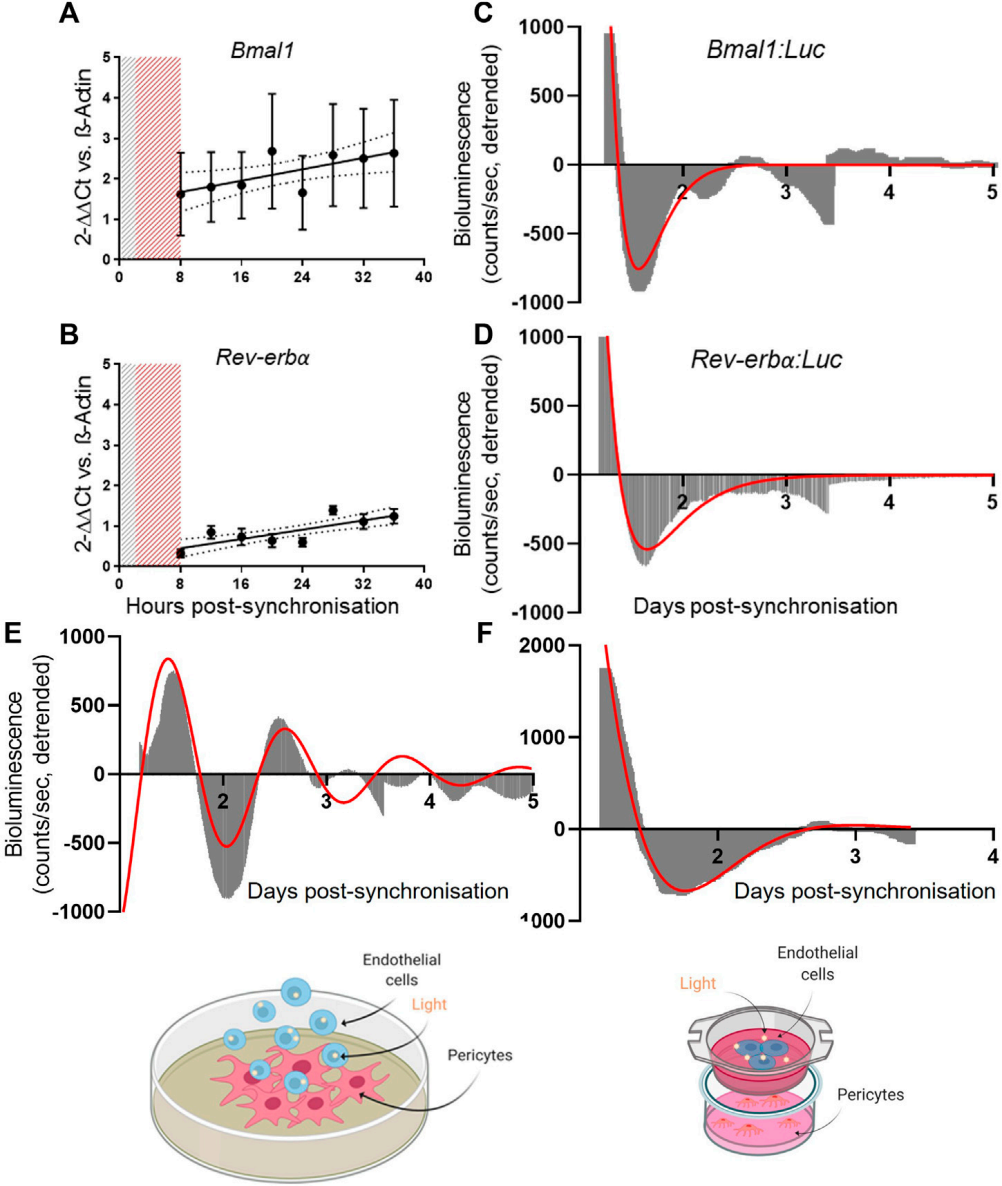
Pericytes’ clock synchronization influences endothelial cells’ Bmal1 rhythmicity. mRNA expression of Bmal1 (N = 3, **(A)**) and Rev-erbα (N = 3, **(B)**) in human umbilical vein endothelial cells (HUVEC). The data are shown as 2^−ΔΔCt^ and normalized on β-actin housekeeping mRNA expression. Error bars represent SEM and 95% CI is shown in the graph. Non-linear regression (Cosinor Curve) was compared against straight line; straight line is the preferred model (p > 0.05). Average detrended non-oscillatory profile of serum shock synchronized HUVEC cells transduced with Bmal1:Luc (N = 2, **(C)**) or Rev-erbα:Luc (N = 2, **(D)**) lentivectors. Data are shown in area graphs and counts/sec are plotted against days post-synchronization. Red line represents a damped sin wave with a period of 24 h. Average detrended oscillatory profile of Bmal1:Luc in HUVEC cells in a contact (N = 3, **(E)**) and non-contact (N = 3, **(F)**) co-culture with synchronized saphenous vein pericytes (SVP) Image shows a schematic of the protocol. Red line represents a damped sin wave with a period of 24 h.

Here, we assessed the clock gene expression in primary human endothelial cells using the same tools applied to pericytes. In our experimental set-up, we were unable to confirm the presence of a circadian clock in HUVECs. Gene expression analysis revealed a non-rhythmic expression of *Bmal1* and *Rev-erbα* (Figures 2A,B; *N* = 3), as shown by the preferred fitted model being a straight line rather than a cosinor (*p* > 0.05). These results were confirmed in the bioluminescence reporter measures of promoter activity where none of the promoters showed rhythmicity (Figures 2C,D; *N* = 2, *n* = 6) We also tried synchronization with dexamethasone rather than serum shock, but this yielded comparable results (Supplementary Figure S1).

Next, we co-cultured endothelial cells (HUVEC) with pericytes (SVP) by seeding *Bmal1*:Luc transduced endothelial cells on top of a synchronized monolayer of pericytes. In this contact co-culture approach, *Bmal1* promoter activity in the endothelial cells showed a 24 h oscillation up to 3 days of culture (Figure 2E; *N* = 3). To assess whether the synchronizing effect was due to contact-dependent factors or secreted molecules exchanged between the two cell types, we repeated the experiment in a non-contact co-culture. *Bmal1*:Luc transduced endothelial cells were seeded on transwell insert, placed on top of a monolayer of synchronized pericytes. We observed that the lack of contact prevented endothelial cells’ clock synchronization, suggesting that the synchronization is at least in part dependent upon contact-mediated mechanisms (Figure 2F; *N* = 3).

These results suggest that, in our experimental conditions, endothelial cells synchronization requires stimuli imparted by pericytes, co-cultured in close contact.

### 3.3 BMAL1 Knockdown in Vascular Cells Reduces Their Angiogenic Potential

Endothelial cells are the main drivers of blood vessel sprouting (angiogenesis), however their function is finely regulated by perivascular cells, such as pericytes (Armulik et al., 2005; Campagnolo et al., 2016; Caporali et al., 2017; Eilken et al., 2017).

**FIGURE 3:**
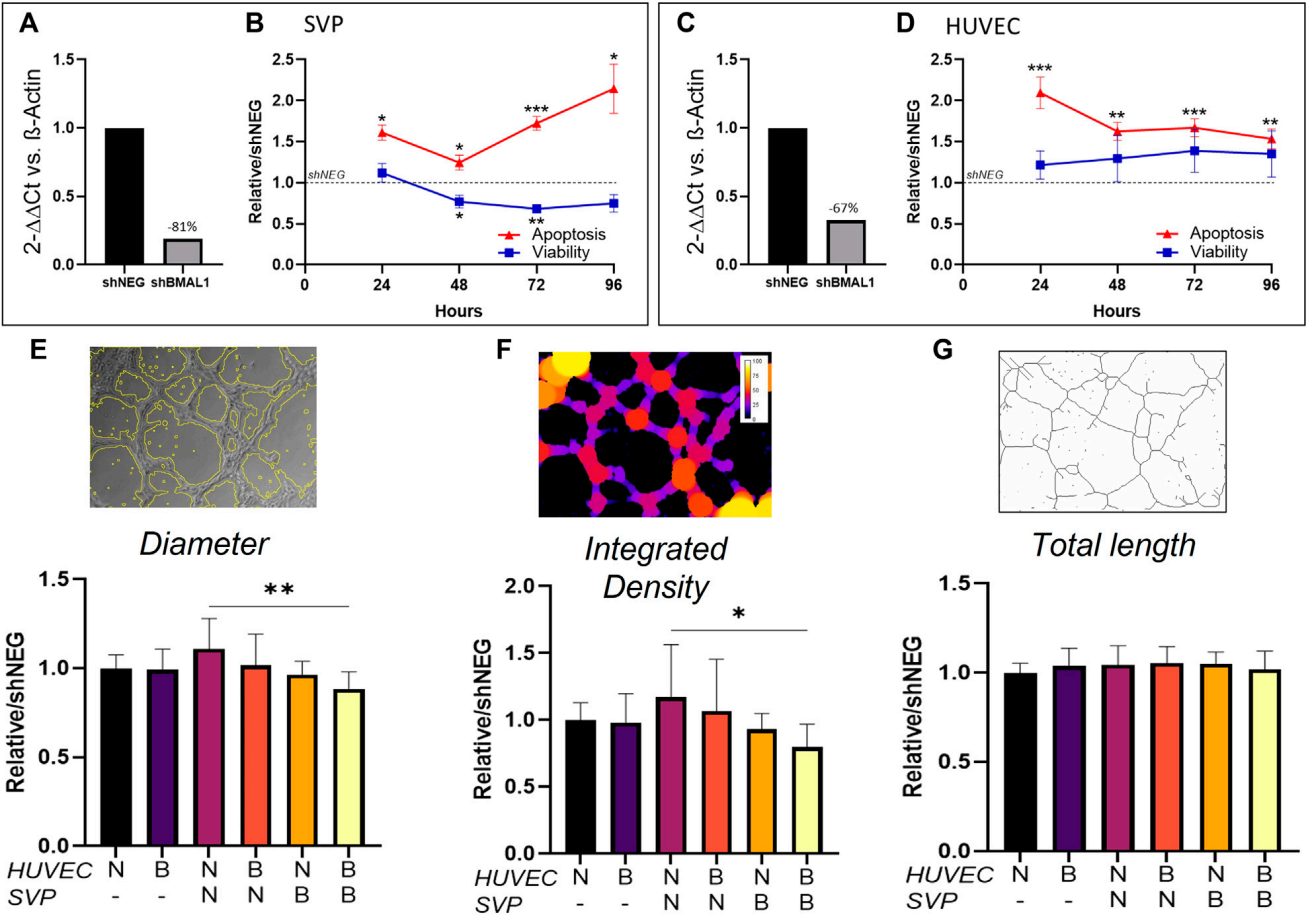
Clock disruption affects tube formation in Matrigel assay. Bmal1 mRNA expression in saphenous vein pericytes (SVP, **(A)**) and in human umbilical vein endothelial cells (HUVEC, **(C)**) in knockdown cells (shBMAL1) in comparison with control cells (shNEG). The data are shown as 2^−ΔΔCt^ and normalized on β-actin housekeeping mRNA expression. Fold change in viability and apoptosis of shBMAL1 cells over time, relative to shNEG cells in SVP (n = 4, **(B)**) and HUVEC (n = 4, **(D)**), respectively. Relative average branch thickness (**(E)**, Diameter), network coverage (**(F)**, Integrated density) and total branch length (**(G)**, Total length) measured in HUVEC (H) cultured alone or in co-culture with SVP (S) on Matrigel. Different combination of shNEG cells (N) and shBMAL1 (B) cells are compared. Representative mask pictures of the analysis are shown. Data were analyzed using two-way ANOVA, and * = p < 0.05, ** = p < 0.01, *** = p < 0.001 vs. shNEG or wild-type co-cultures.

Here, we aimed at assessing the effect of the disruption of the vascular clock in endothelial cells and pericytes. We produced BMAL1 knockdown cells by transducing pericytes and endothelial cells with shBMAL1, followed by puromycin clonal selection and obtained 67 and 81% reduction of BMAL1 mRNA expression, respectively (Figures 3A,C). We then verified whether the absence of BMAL1 affected viability and apoptosis. In SVP, BMAL1 knockdown impaired viability compared to controls after 48 h (0.77 ± 0.05; *p* < 0.05) and 72 h (0.68 ± 0.04; *p* < 0.01), linked with a significant increase in apoptosis at all time points (Figure 3B; 24 h: 1.61 ± 0.10, *p* < 0.05; 48 h: 1.24 ± 0.05, *p* < 0.05; 72 h: 1.72 ± 0.05, *p* < 0.001; 96 h: 2.14 ± 0.15, *p* < 0.05). In HUVEC, BMAL1 knockdown did not affect the overall viability of the cells over time but increased apoptosis at all time points (Figure 3D; 24 h: 2.09 ± 0.12, *p* < 0.001; 48 h: 1.62 ± 0.8, *p* < 0.01; 72 h: 1.67 ± 0.06, *p* < 0.001; 96 h: 1.53 ± 0.08, *p* < 0.01).

Next, we assessed the effect of the loss of BMAL1 on angiogenesis by co-culturing combinations of HUVEC ± shBMAL1 with or without SVP ± shBMAL1, to form tube-like structures in a Matrigel assay. Results showed that BMAL1 knockdown in endothelial cells cultured alone or with pericytes did not affect tube formation, however, when both HUVEC and SVP’s clocks were disrupted at the same time, a reduction in the thickness of the branches and the total coverage was observed (Figures 3E,F). Interestingly, the total branching length was not affected in any of the tested conditions (Figure 3G) suggesting that a disrupted clock may only affect the maturation and morphology of the vascular-like structures *in vitro,* rather than the overall angiogenic capacity.

In summary, these results indicated that pericytes are disproportionately affected by circadian disruption and that only the concurrent disruption of clock in both endothelial cells and pericytes determines defective angiogenesis, by reducing vessel maturation.

### 3.4 Lactate Concentration Is Dependent on Pericyte’s Clock Genes in Co-culture

The metabolic by-product lactate is a known intracellular signaling molecule influencing angiogenesis, and is rapidly shuttled between cells, favoring endothelial progenitor cells recruitment, endothelial cells migration and the release of vascular endothelial growth factor (VEGF) and transforming growth factor-beta (TGFβ) (Brooks, 2009; Baltazar et al., 2020; Brooks, 2020). Lactate has been implicated in the regulation of the circadian clock (Rutter et al., 2001; Krishnaiah et al., 2017) and is readily up-taken and utilized by endothelial cells (Harjes et al., 2012).

**FIGURE 4:**
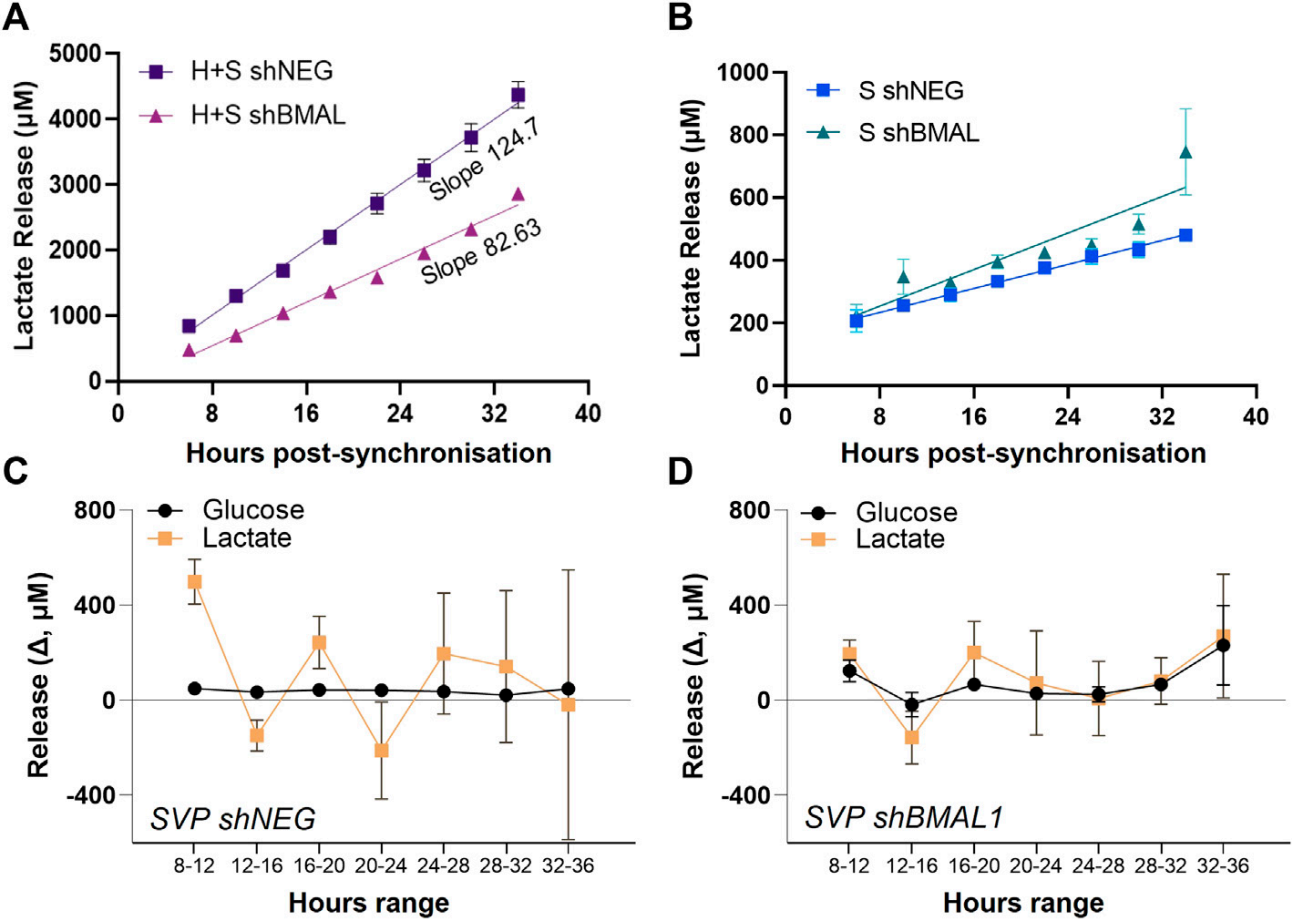
Pericytes’ clock influences endothelial cells’ lactate release. Lactate accumulation in the supernatants of saphenous vein pericytes (SVP, S), transduced with control lentivirus (shNEG) or shBMAL1, and either co-cultured with wild-type human umbilical endothelial cells (HUVEC, H) **(A)** or cultured alone **(B)**. Linear regression is over-imposed. Incremental change (Δ) in lactate and glucose concentration in the supernatants of SVP shNEG **(C)** and SVP shBMAL1 **(D)**, relative to previous timepoint.

We quantified the amount of lactate released in HUVEC/SVP co-cultures after synchronization and detected a steady accumulation of lactate over time in the supernatant (Figure 4A). When BMAL1 was knocked down only in SVP, we observed a decreasing slope in lactate release in the co-culture (Figure 4A; HUVEC + SVP shNEG linear regression slope = 124.7 ± 4.9; HUVEC + SVP shBMAL1 linear regression slope = 82.63 ± 2.6), associated with a lower concentration of lactate in the supernatant at all time points (mean difference 163 ± 81 µM). This is remarkable, especially considering that endothelial cells are the major producers of lactate within the co-culture (Supplementary Figure S2).

Of note, single culture of pericytes produced and released lactate, even if in lower concentrations as compared to endothelial cells. Disruption of the circadian clock in SVP did not dramatically impact the overall lactate accumulation overtime (Figure 4B); however shNEG pericytes produced lactate with an oscillatory pattern with a period of approximately 8 h (Figure 4C), while shBMAL1 pericytes did not display an oscillatory pattern (Figure 4D). These changes were specific to the lactate, as glucose remained constant throughout (Figures 4C,D).

In conclusion, we demonstrated that clock disruption in pericytes affects the rhythmicity of lactate production and influences the overall accumulation of lactate in co-cultures. This may constitute a mediator influencing endothelial cells’ clock synchronization and angiogenesis.

### 3.5 Vascular Clock Synchronization Promotes the Formation of Complex Vascular-like Structures in a 3D Tissue Engineered Scaffold

Vascularization of tissue engineering constructs is critical to the development of complex 3D structures, however the role of the circadian clock in this process has never been investigated.

**FIGURE 5:**
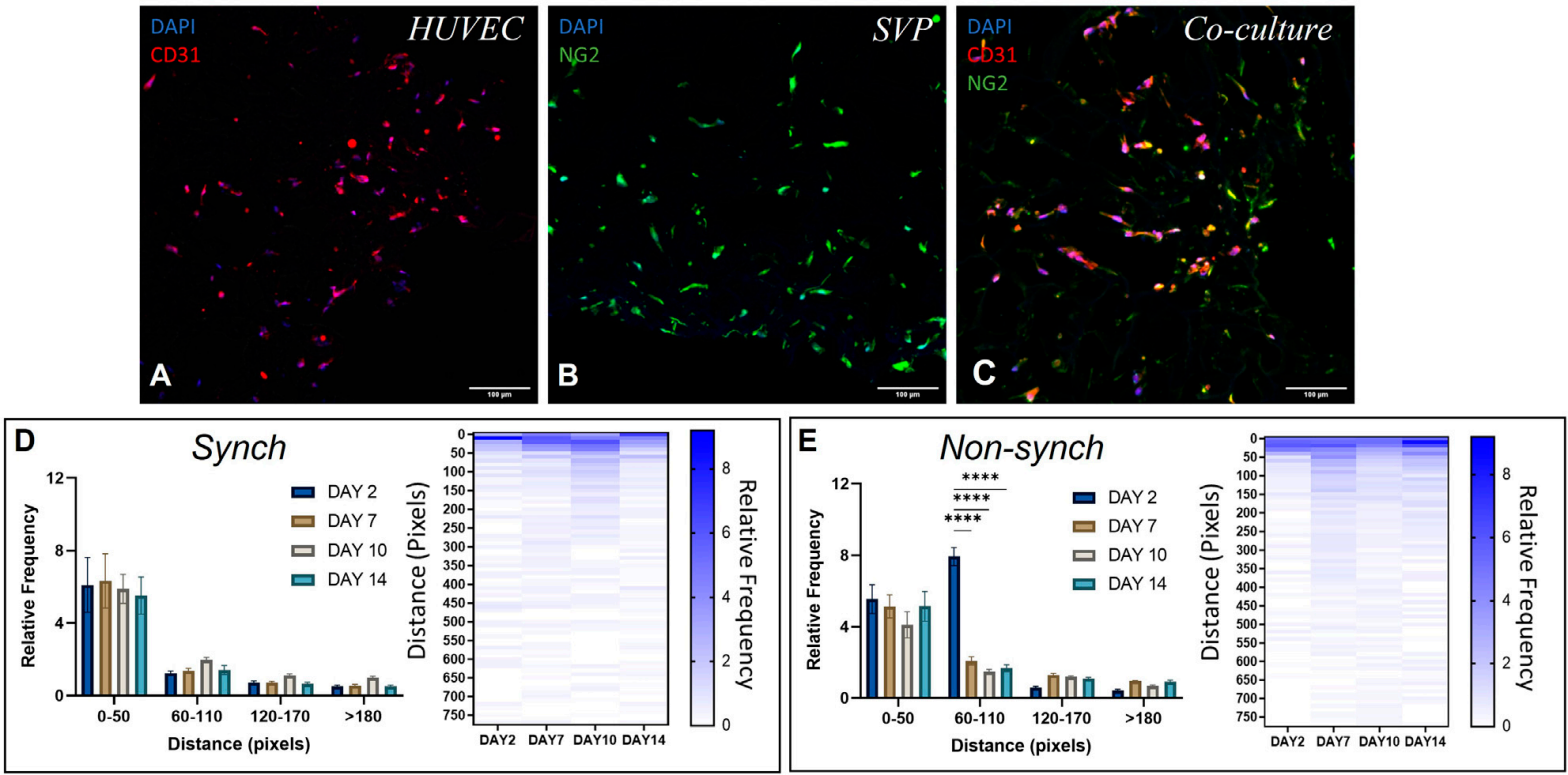
Distribution of endothelial cells and pericytes in a 3D polyurethane scaffold. Representative immunofluorescence images of scaffolds seeded with human umbilical vein endothelial cells (HUVEC, **(A)**), saphenous vein pericytes (SVP, **(B)**) and a co-culture of HUVEC and SVP **(C)**. CD31 (red), NG2 (green) and DAPI (blue). Scale bar:100 µm. Quantification of relative cell distribution in the scaffold, in synchronized (synch, **(D)**) and non-synchronized (non-synch, **(E)**) co-cultures. Heat maps show the relative frequency up to 750 pixels of distance. Data were analyzed using two-way ANOVA, and **** = p < 0.0001.

HUVEC and SVP were seeded separately in a polyurethane scaffold coated with fibronectin and gelatin, which provided a structured three-dimensional microenvironment with extracellular matrix (ECM) mimicry, offering a biomimetic biochemical composition for the cells to interact with the ECM and each other (Totti et al., 2018). We observed that both HUVEC (CD31^+^ cells) and SVP (NG2^+^ cells) were able to penetrate, adhere and grow inside the scaffold (Figures 5A,B).

We then seeded both cell types together in co-culture and observed marked changes in the morphology of cell distribution within the scaffold. In particular, vascular cells became more organized and formed vessel-like structures by lining the scaffold pores (Figure 5C).

To assess whether synchronization of the circadian clocks improved cell migration within the scaffold, we synchronized the cells within the scaffolds with a single pulse of dexamethasone and thereafter cultured the scaffolds for 2, 7, 10 and 14 days post-synchronization. We calculated the relative frequency distribution of cells relative to the distance from the edge of the scaffold. In both synchronized (synch) and non-synchronized (non-synch) conditions we observed a predominant presence of cells in the range 0–50 pixels from the edge, at every time point (Figures 5D,E, N = 3).

**FIGURE 6:**
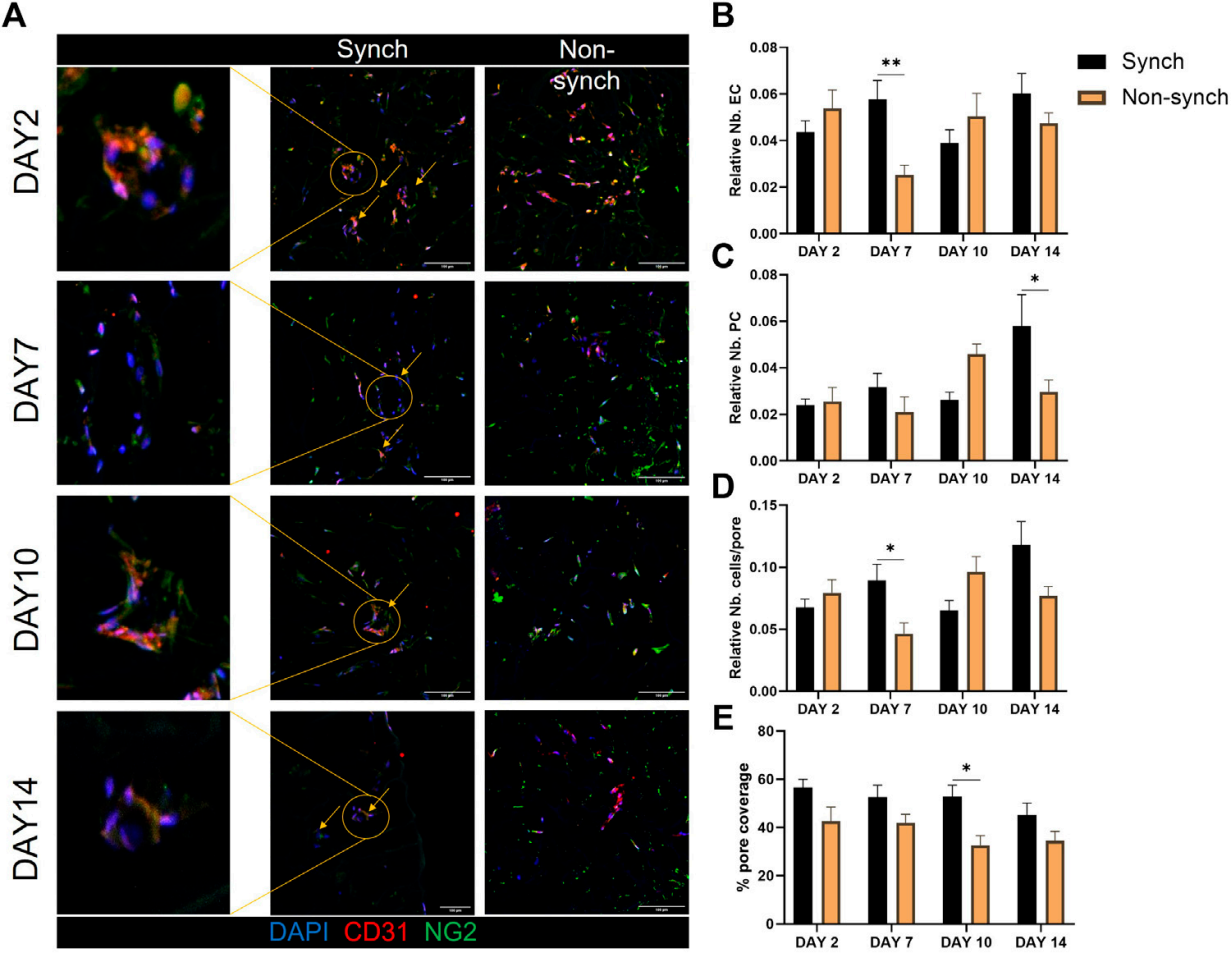
Clock synchronization improves vascular structure formation in 3D. Representative immunofluorescence images of synchronized (Synch) and non-synchronized (Non-synch) co-cultures on poly-urethane scaffolds, at different time-points [**(A)**, N = 3 for each time point]. Endothelial cells (CD31, red) and pericytes (NG2, green), nuclei in blue (DAPI). Scale bars: 100 µm Quantification of the number of endothelial cells **(B)** and pericytes **(C)** relative to the total number of cells in each pore, total number of cells per pore **(D)** and percentage of pore coverage **(E)** was compared in Synch and Non-synch conditions. Two-way ANOVA, * = *p* < 0.05, ** = *p* < 0.01 vs. Non-synch at each time-point.

On the other hand, we noticed an increase in vessel-like structures associated with a better/more systematic organization of cells around the pores in the synchronized clock condition, at every time point of culture (Figure 6A). We quantified the average number of endothelial cells and pericytes in each pore and identified a significant increase of endothelial cells that associated with the scaffold pores at day 7, and of pericytes at day 14 and an overall increase in the number of cells per pore at day 7 (Figures 6B–D). Additionally, we calculated the pore coverage, as the percentage of the pore surface covered with cells, identifying a trend of increased coverage throughout the culture in synchronized samples, compared with non-synchronized samples (Figure 6E). Specifically, the mean percentage of pore coverage in the synchronized condition was always higher (53.3% ± 8.4) when compared with the non-synchronized condition (36.2% ± 10.4), with a significant difference at 10 days of culture (*p* < 0.05).

Taken together these results indicate that circadian clock synchronization affects the organization of vascular cells in complex vascular structures within a 3D scaffold.

## 4 Discussion

During the angiogenic process, the crosstalk between endothelial cells and pericytes is of paramount importance for the formation of a mature and organized vascular network (Gerhardt and Betsholtz, 2003; Cathery et al., 2018). A new insight into this cross-communication was presented in this manuscript. We demonstrated that pericytes possess an endogenous self-sustained molecular clock, while endothelial cells did not display a rhythmicity in our experimental settings, opening up questions on the potential clock-to-clock communication. Indeed, we found that synchronized pericytes can “start” the endothelial cells’ clock in contact co-culture. Moreover, when the clock was disrupted in both pericytes and endothelial cells by *Bmal1-*targeting shRNA, pericytes were overwhelmingly more affected in terms of viability and apoptosis. In an angiogenesis assay, the maturation of vessel-like structures was impaired when both endothelial cells and pericytes were lacking *Bmal1* expression. This was not the case when the clock was disrupted in only one cell type, suggesting a functional redundancy between the clocks in these cells, which may explain why HUVEC circadian rhythms are absent or low amplitude when cultured as a mono-culture. We also identified a potential implication of the metabolic by-product lactate in the vascular cells’ crosstalk, showing that a disrupted clock in pericytes disproportionately affected lactate accumulation in the co-culture supernatants, possibly due to the disruption of the rhythmic accumulation of lactate produced by the pericytes and a knock-on effect on endothelial cells clock and lactate production/usage. Furthermore, we cultured endothelial cells and pericytes in a 3D macroporous polyurethane scaffold and demonstrated how a synchronized vascular clock drives a better organization of vascular structures in a 3D micro-environment.

Despite the vast knowledge regarding vascular function and circadian rhythms, further evidence is needed to clarify the role of perivascular cells (pericytes and smooth muscle cells). Several papers presented *ex vivo* evidence of the rhythmicity of the vascular wall (Yoo et al., 2004; Anea et al., 2009), and given the preponderance of vascular smooth muscle cells in large vessel wall as compared to endothelial cells, it is likely that the effect might have been driven mostly by perivascular cells. Endothelial specific *Bmal1*-KO animal experiments have shown an accelerated retinal microvascular and femoral arterial macrovascular injury (Bhatwadekar et al., 2017), and an altered pattern of diurnal variation in the time to thrombotic vascular occlusion (TTVO) (Westgate et al., 2008). Hence, endothelial cells possess an endogenous clock *in vivo*, but whether the clock is endogenously regulated or paced by external signals is more debated. In our experimental set-up, human umbilical vein endothelial cells cultured *in vitro* did not express a circadian rhythmicity of clock genes, suggesting that while they possess an endogenous circadian clock, it requires exogenous stimulation to be fully expressed. Westgate *et al.* also reported that endothelial cells extracted from mice and cultured *ex vivo* failed to express circadian oscillations (Westgate et al., 2008). On the other hand, Takeda et al. reported the existence of a molecular clock in HUVEC cells, although the amplitude of the oscillation resulted lower when compared to hemangioendothelioma cells (Takeda et al., 2007). It is conceivable, therefore, that at least *in vitro*, the endothelial cells circadian rhythmicity may depend on external signals.

Here, we demonstrated that pericytes, essential components of the capillaries, can rhythmically express clock genes. Moreover, the protein levels of BMAL1 and REV-ERB in the nuclei followed an oscillatory pattern, where the peak accumulation was observed approximately 8 h after the mRNA expression peak. Bioluminescence data also showed a rhythmicity in the promoter activity patterns.

In the native microvasculature, pericytes lay in close physical contact with endothelial cells, surrounding the abluminal endothelial conduit, and forming physical junctions. These specialized junctions mediate the cell-to-cell contact through contractile forces and the passage of metabolites between cells (Allt and Lawrenson, 2001; Armulik et al., 2005; Geevarghese and Herman, 2014). Pericytes contribute to the endothelium functionality *in vivo*, including homeostasis and permeability, regulating endothelial cells proliferation and differentiation, and providing vascular contractility and tone (Gaengel et al., 2009; Mastrullo et al., 2020). They are also capable of sensing and responding to physiological signals, such as the angiotensin II, endothelin-1, PDGF and oxygen levels (Rucker et al., 2000; Bergers and Song, 2005), which are known to be regulated in a circadian fashion. We therefore investigated whether a synchronized clock in pericytes could serve as a stimulus for the downstream synchronization of endothelial cells. Pericytes-endothelial crosstalk involves both contact-mediated and paracrine communication, thus we tested the influence of pericyte synchronization in two co-culture settings, allowing to discriminate between the two types of communication. We observed that in the contact co-culture, synchronized pericytes were able to drive endothelial cell synchronization. This was not seen in non-contact co-cultures, suggesting that endothelial synchronization by pericytes requires proximity.

We speculate that lactate could be one of the factors involved in the clock’s crosstalk. Lactate is a product of the metabolism with a short half-life, and it is known to mediate both cell-cell and intracellular signaling exerting autocrine-, paracrine- and endocrine-like actions (Brooks, 2009). Interestingly, addition of lactate to neuroblastoma cells stimulates NPAS2/BMAL1 activity (Rutter et al., 2001) but its release only showed a rhythmicity in response to BMAL1 knockdown in human bone osteosarcoma epithelial cells (U2OS) cells (Krishnaiah et al., 2017). Here, we showed that clock disruption in pericytes impaired the biological oscillation of lactate production. In co-culture, the accumulation of lactate was dramatically reduced when BMAL1 was knocked-down in pericytes. However, endothelial cells are the main source of lactate in the co-culture, hence suggesting that changes in lactate oscillation in pericytes affects the endothelial cell utilization and/or production of the metabolite, indicating that lactate could act as a cell-cell signal in this process. This hypothesis is in line with previously published work suggesting that intracellular lactate can be shuttled between different cell types in highly metabolically active organs such as the heart (fibroblasts to cardiomyocytes) (Rakus et al., 2016) and the brain (astrocytes to neurons) (Mason, 2017), and function as a cell-cell signal. The detected changes in extracellular levels of lactate may reflect changes in the intracellular lactate, and therefore suggest a signaling role for intracellular lactate, explaining the requirement for cell-cell contact in the entrainment of endothelial cells by pericytes.

Circadian disruption has been associated with pericyte dysfunction in mice and with unhealthy and uncontrolled angiogenesis in zebrafish and mice models (Jensen et al., 2014; Nakazato et al., 2017). *In vivo*, the process of angiogenesis is profoundly regulated by the interaction between endothelial cells and pericytes and the 3D microenvironment in which they are embedded (Jensen et al., 2014). For this reason, we investigated the role of the clock disruption in 3D using *in vitro* angiogenic assays. We demonstrated that a disrupted clock in pericytes and endothelial cells impairs the capability of forming mature vessel-like structures in a 3D angiogenesis assay (Matrigel). We cannot exclude that this effect could in part be due to the reduced viability observed in knockdown SVP, however it is interesting that the change in angiogenic features is only observed when BMAL1 knockdown was performed on both cell types.

The formation of a connected network of vascular structures is particularly critical for the successful creation of tissue engineering constructs, and the definition of the factors regulating the correct development and maturation of vessels within a 3D scaffold is of utmost importance in the field (Mastrullo et al., 2020). Furthermore, ECM-modified synthetic porous scaffolds provide a very promising 3D environment for the natural organization of vascular cells (Gupta et al., 2020). For this reason, we furthered our findings by culturing endothelial cells and pericytes either alone or in combination in 3D macroporous tissue engineering polyurethane scaffolds. As expected, co-cultures thrived and were characterized by a complex organization of cells around the existing pores of the scaffolds. Previous studies reported the formation of vascular networks in 3D tissue engineering scaffolds from HUVEC and pericytes (Sathy et al., 2015; Parthiban et al., 2020), here we crucially demonstrated that the circadian clock synchronization supported a further maturation of more complex and organized vascular structures. Disappointingly, despite several studies showing migration of endothelial cells inside tissue engineering scaffolds (Ahmed et al., 2018; Landau et al., 2018), we observed that most of the cells did not penetrate deeply inside our scaffold core. Crucially, Koo et al. reported that HUVEC could migrate inside the scaffold only by applying flow perfusion, due to their change in shape in response to shear stress (Koo et al., 2014). On the other hand, Gupta et al. observed a good migration of immortalized human microvascular endothelial cells (HMEC) inside an ECM-modified scaffold over 28 days of culture (Gupta et al., 2020). Hence, the behavior of cells on this specific scaffold might be due to the scaffold’s characteristics of porosity and pore connectivity (Totti et al., 2018), or might be cell specific.

In summary, our results provide novel insights on the importance of circadian clock in the development of new capillaries during regenerative angiogenesis, and in the context of tissue engineered constructs. Critically, we show a high level of contact-based co-regulation between redundant clocks in pericyte and endothelial cells is required for healthy angiogenesis in culture. Indeed, several pericyte populations have been used in regenerative medicine, including the SVP cells used in this work (Katare et al., 2011; Alvino et al., 2018), to stimulate reparative angiogenesis. Furthermore, pericytes are an attractive source of cells for tissue engineering due to their progenitor nature, and their ability to stimulate angiogenesis and tissue repair (Mastrullo et al., 2020). Our work suggests that the synchronization of the circadian clock within and between cell populations should become a factor to consider when developing a new regenerative medicine approach or designing a tissue engineering construct.

## Data Availability

All raw data, images, and analyses supporting the findings of this study are available through the online repository Zenodo (https://doi.org/10.5281/zenodo.6323605).
