# Supplementary material for: Pericytes’ Circadian Clock Affects Endothelial Cells’ Synchronization and Angiogenesis in a 3D Tissue Engineered Scaffold
Source: Front Pharmacol. 2022 Mar 21;13:867070. doi: 10.3389/fphar.2022.867070 (PMC8977840; doi:10.3389/fphar.2022.867070)
Supplement: Supplementary file 1 [file Table1.DOCX]

Table 1. List of primers used for mRNA expression of clock genes in human cells.

| Gene | GenBank | Forward primer (5’-3’) | Reverse Primer (5’-3’) |
| --- | --- | --- | --- |
| Bmal1 | NM_001030272 | TGGATGAAGACAACGAACCA | TAGCTGTTGCCCTCTGGTCT |
| Clock | NM_001267843 | GCAGCAGCAGCAGCAGAG | CAGCAGAGAGAATGAGTTGAGTTG |
| Per2 | NM_022817 | GACATGAGACCAACGAAAACTGC | AGGCTAAAGGTATCTGGACTCTG |
| Rev-erbα | NM_021724 | TGCTGCAGGGTGCTTCGGAT | TAGGTGATGACGCCACCTGTGT |
| β-Actin | NM_001101.4 | AGAGCTACGAGCTGCCTGAC | AGCACTGTGTTGGCGTACAG |
