# Supplementary material for: Pericytes’ Circadian Clock Affects Endothelial Cells’ Synchronization and Angiogenesis in a 3D Tissue Engineered Scaffold
Source: Front Pharmacol. 2022 Mar 21;13:867070. doi: 10.3389/fphar.2022.867070 (PMC8977840; doi:10.3389/fphar.2022.867070)
Supplement: Supplementary file 2 [file Table2.DOCX]

Table 2. List of primers used for the purification of human promoters from human DNA.

| Gene | Forward primer (5’-3’) | Reverse Primer (5’-3’) |
| --- | --- | --- |
| Bmal1 | ACCCAGAGAAGAGGGACATC | CTCCGTCCCTGACCTACTTT |
| Per2 | TGAGGGCGTAGTGAATGGAAG | TGTCACCGCAGTTCAAACGA |
| Rev-erbα | ATCTACATGTTCCCCTCTGAGTAGT | TATTTCACTCTGCCAATCTCAGCC |
