# Supplementary material for: Pericytes’ Circadian Clock Affects Endothelial Cells’ Synchronization and Angiogenesis in a 3D Tissue Engineered Scaffold
Source: Front Pharmacol. 2022 Mar 21;13:867070. doi: 10.3389/fphar.2022.867070 (PMC8977840; doi:10.3389/fphar.2022.867070)
Supplement: Supplementary file 3 [file DataSheet1.DOCX]

Supplementary Material

# Supplementary Figures


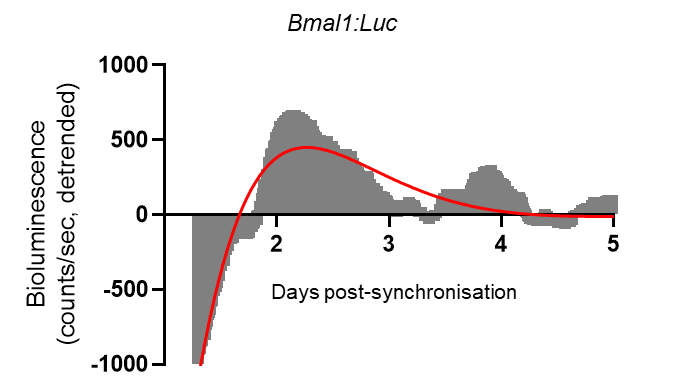


Supplementary Figure 1. *Bmal1*:Luc promoter activity in endothelial cells following dexamethasone synchronization. *Average detrended non-oscillatory profile of dexamethasone synchronized human umbilical vein endothelial cells (HUVEC) transduced with Bmal1:Luc (n=1) lentivectors. Data are shown in area graphs and counts/sec are plotted against days post-synchronization. Red line represents a damped sin wave with a period of 24h.*


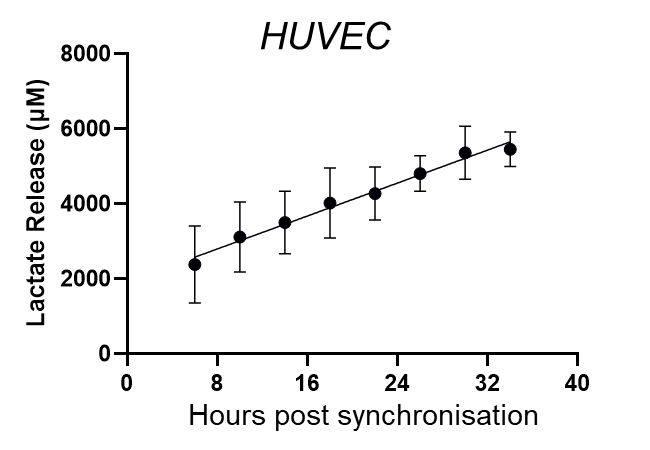


Supplementary Figure 2. Lactate release in human umbilical vein endothelial cells (HUVEC) supernatants. *Lactate accumulation in the supernatants of HUVEC cultures over time (hours post-synchronization). Linear regression is over-imposed.*
